# Predictive Power of Cognitive Biomarkers in Neurodegenerative Disease Drug Development: Utility of the P300 Event-Related Potential

**DOI:** 10.1155/2022/2104880

**Published:** 2022-11-08

**Authors:** John Olichney, Jiangyi Xia, Kevin J. Church, Hans J. Moebius

**Affiliations:** ^1^Center for Mind and Brain, University of California, Davis, Davis, CA, USA; ^2^Department of Neurology, University of California, Davis, Sacramento, CA, USA; ^3^Athira Pharma, Inc., Bothell, WA, USA

## Abstract

Neurodegenerative diseases, such as Alzheimer's disease (AD), and their associated deterioration of cognitive function are common causes of disability. The slowly developing pathology of neurodegenerative diseases necessitates early diagnosis and monitored long-term treatment. Lack of effective therapies coupled with an improved rate of early diagnosis in our aging population have created an urgent need for the development of novel drugs, as well as the need for reliable biomarkers for treatment response. These issues are especially relevant for AD, in which the rate of clinical trial drug failures has been very high. Frequently used biomarker evaluation procedures, such as positron emission tomography or cerebrospinal fluid measurements of phospho-tau and amyloid beta, are invasive and costly, and not universally available or accessible. This review considers the functionality of the event-related potential (ERP) P300 methodology as a surrogate biomarker for predicting the procognitive potential of drugs in clinical development for neurocognitive disorders. Through the application of standardized electroencephalography (EEG) described here, ERP P300 can be reliably measured. The P300 waveform objectively measures large-scale neuronal network functioning and working memory processes. Increased ERP P300 latency has been reported throughout the literature in disorders of cognition, supporting the potential utility of ERP P300 as a biomarker in many neurological and neuropsychiatric disorders, including AD. Specifically, evidence presented here supports ERP P300 latency as a quantitative, unbiased measure for detecting changes in cognition in patients with AD dementia through the progression from mild to moderate cognitive impairment and after drug treatment.

## 1. Introduction

Neurodegenerative diseases, such as Alzheimer's disease (AD), Parkinson's disease (PD), and Huntington's disease (HD), can lead to dementia, which affects nearly 55 million people worldwide [1]. The neurodegenerative processes start decades before the appearance of clinical symptoms [2–6]. After diagnosis, the clinical course and life expectancy of patients with chronic neurodegenerative conditions often span many years, necessitating the long-term use of therapeutics. However, the progressive nature of neurodegenerative diseases poses a significant challenge for drug development. As the result of budgetary constraints and risk considerations, many early translational trials of drugs for these diseases had to be limited in size and/or duration [7]. Reliable cognitive assessment is particularly difficult because of placebo response and slow clinical decline. Longer trials (≥6 months) are usually necessary to allow for separation of trial drug effects from a placebo arm [2, 8]. Additionally, a leading cause of failure of central nervous system (CNS) drugs in clinical trials is use of the incorrect dose, which may only become apparent during phase 3 trials [9, 10]. Furthermore, the disease may be too progressed in patients with neurodegenerative diseases often selected for clinical trials [9]. Therefore, the exceptionally high failure rate of clinical trials for AD, coupled with the increasing rate of diagnosis has led to an urgent need for the development of novel therapeutics [7]. Despite this demand, the methods available for early prediction of clinical outcomes following pharmacological interventions are limited. Methods such as positron emission tomography are cost-prohibitive, and the cerebrospinal fluid measurement of phospho-tau and amyloid beta is invasive. Furthermore, these methods have limited predictive value [11]. Together, these factors underscore the need for objective, quantitative, reliable, noninvasive, repeatable, and cost-effective approaches for the assessment and monitoring of cognitive status. This review explores the ability of recording neurophysiological activity using the event-related potential (ERP) P300 to serve as a biomarker of cognition in clinical trials. The review focuses on the P300 component of the ERP waveform, including important technical aspects of the methodology, and summarizes the existing evidence of its translational utility in evaluating potential procognitive properties of pharmaceutical interventions in AD, other neurodegenerative diseases, and neuropsychiatric disorders.

## 2. Event-Related Potentials (ERPs)

**Figure 1:**
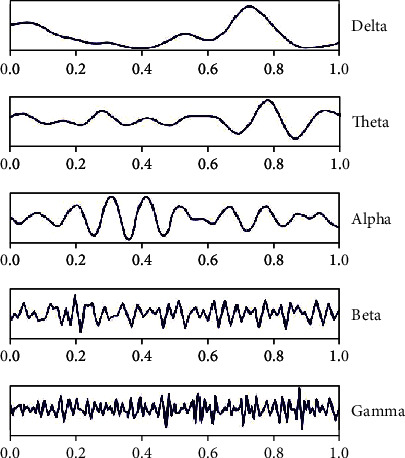
Quantitative electroencephalography (qEEG). qEEG is a reliable measure of neurophysiological electric activity. qEEG measures activity of the brain, usually in the resting state, and is sensitive to changes in synaptic function and network connectivity [12]. The five distinct frequency bands that can be analyzed with qEEG have unique brain function associations: delta (<4 Hz) is associated with deep sleep; theta (4–8 Hz) correlates with rapid eye movement (REM) sleep and quiet wakefulness, as well as aspects of memory and language processes; alpha (8–14 Hz) is related to suppression of task-irrelevant activity during “idle” cognitive processing; beta (14–30 Hz) is associated with arousal, alertness, and voluntary movement; and gamma (>30 Hz) is most relevant to cognitive function and attention. The effects of drug candidates on qEEG lend themselves ideally to detect changes in the saliency network, which is particularly useful in neurodegenerative and neuropsychiatric conditions with cognitive impairment [124]. Furthermore, qEEG is translatable from animal models of disease to humans; hence, qEEG can be used as the first step in narrowing the dose of a drug by correlating the pharmacokinetics and pharmacodynamics of the drug to changes in qEEG [14]. For example, auditory EPs can be used to correlate antidepressant and antipsychotic responsiveness in animal models to humans [14]. Correlating qEEG changes with drug doses can lessen the need to define doses based on psychometric outcomes and the associated risk; defining these doses typically requires larger and longer clinical trials, and results are highly variable.

ERPs and evoked potentials (EPs) are particularly useful applications of electroencephalography (EEG), in that they capture averaged brain responses that are task-based and time-locked and thus, are associated with specific sensory and motor EPs or cognitive events. As a task-based methodology, and thus distinct from non–task-based quantitative electroencephalography (qEEG; Figure 1), the ERP is a highly suitable measure for correlating neuronal function with cognitive processes, such as working memory and executive function. The ERP waveform, which is composed mainly of summated inhibitory and excitatory postsynaptic potentials, measures the activity of networked neurons firing in synchrony, or integrated synaptic activity [3, 12]. Thus, ERPs can provide a neural correlate of working memory load and quantify other higher-level cognitive processes [3]. The ERP measurement is particularly appropriate for the evaluation of synaptic disorders, such as AD, because it reflects the spatial processing speed within large neuronal networks [12]. The superior temporal resolution of ERPs makes them well suited to detecting neural response to task manipulations (in milliseconds) and to distinguishing and quantifying both the early (generally ≤200 ms) and later stages of cognitive processing in individuals, including those with mild-to-moderate stage dementia [12, 13]. Thus, the superior temporal resolution of ERPs enables pharmacodynamic changes to be detected within short time frames and allows the results to be compared with baseline data [14]. Capturing changes in cognitive function utilizing ERPs may serve as a promising noninvasive biomarker that may increase confidence in the detection of procognitive effects of drugs during later stages of development.

**Figure 2:**
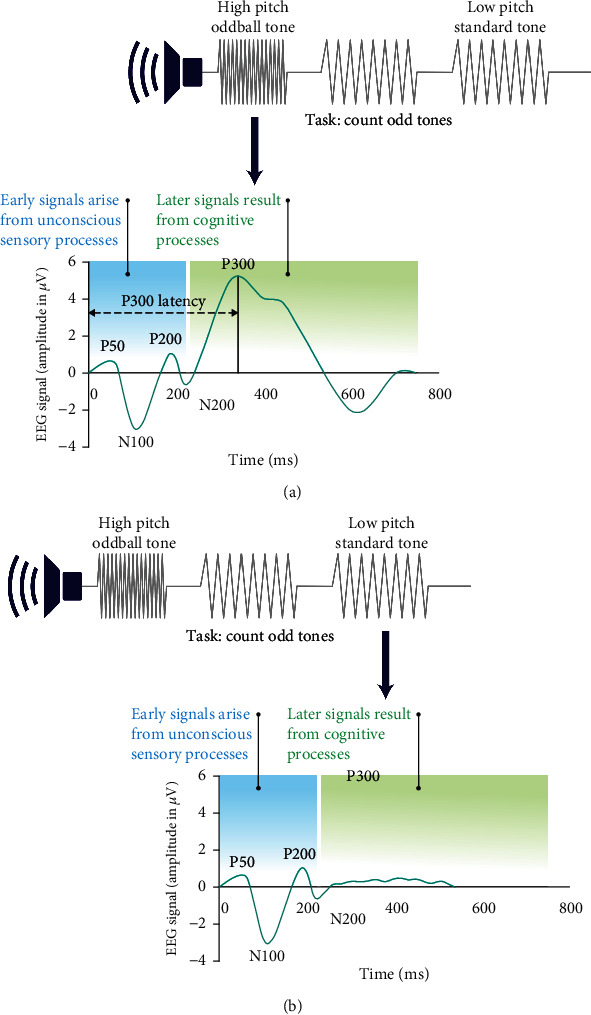
Schematic of the ERP P300 in the auditory oddball paradigm. The auditory oddball paradigm consists of a sequence of audio tones with randomized presentations of frequent (e.g., low-pitch [500-Hz] standard stimulus tones) and rare (e.g., high-pitch [2000 Hz]) “oddball” tones, with interstimulus intervals (1.2–1.9 s). Participants are tasked with counting the number of oddball tones (a). During the task, participants' brain activity is recorded with electrodes placed on the scalp. Brain waves are amplified, digitized, and filtered to produce an average waveform with positive (P) and negative (N) deflections: P50, P200, P300, N100, N200 amplitudes, and latencies. The brain produces a large positive deflection 300 ms (P300) after the presentation of an oddball tone, corresponding to recognition of the different stimuli and indicating the activity of the working memory. The amplitude and latency of the P300 can be compared between groups. (b) When a standard tone is presented, no large positive deflection is produced at 300 ms, indicating that the tone is not registered in the working memory.

The deflections that occur within the ERP waveform depend on the task or stimuli presented. The early waves are most closely associated with sensory processing of a presented stimulus and usually occur within ~100 ms of stimulus onset [15]. The later stages of the ERP signal, i.e., those occurring after 200 ms, correspond to controlled attention, working memory access, and integrative processing (e.g., semantic and emotional) of the stimulus. The N component of the ERP waveform refers to the negative voltage deflection, whereas the P component refers to the positive voltage peak (Figure 2).

The number following N or P refers to the typical time from presentation of a stimulus to the peak or deflection (in milliseconds), or latency.

The P50 waveform is an early positive peak elicited by paired-click or steady-state paradigms [15] related to "sensory gating" that has been useful in studies of bipolar disorder and schizophrenia [16, 17]. The N100 and P200 waveforms represent the automatic sensory process responses elicited by auditory stimuli, whereas the N140 and N170 waveforms are elicited by visual stimuli. The P300 waveform appears in response to active engagement in the detection of task-relevant target stimuli. The N200 waveform, and associated “mismatch negativity,” is an additional late response to an infrequent or unexpected stimulus that is often studied in conjunction with the P300 waveform [3]. Additional late components of the ERP—the N400 and P600 waveforms—are elicited in language-processing contexts, such as semantic incongruity and syntax error processing. A late positive component that also has a positive peak at approximately 600 ms following stimulus onset has been shown to be one of the best predictors of human verbal memory ability [18–20].

The P300 component of the ERP waveform, which is considered a later-stage ERP signal, is the most widely analyzed ERP in cognitive research. The P300 component is characterized by the time it takes for the peak to occur (latency), normally ~300 ms after stimulus onset, and by its amplitude. The P300 peak latency reflects the timing of brain activity associated with accessing working memory and performing higher executive functions. The P300 component can be broken down further into subcomponents. The P3a subcomponent occurs in response to distracter (task-irrelevant) stimuli (e.g., a dog barking) and is associated with attentional processes involved in automatic novelty detection. The P3b subcomponent is evoked by target (task-relevant) stimuli [21]. In normal aging, age-related amplitude reduction, latency prolongation, and topographically more frontally oriented P300 have been consistently reported across studies [22]. Decreases in P300 amplitude and increases in latency more severe than those in normal aging have been reported in patients with dementia, psychiatric disorders, alcohol dependence, and traumatic brain injury (TBI), and in neurodevelopmental disorders [23–25]. The utility of the P300 latency as an objective measure in detecting changes in the cognitive performance for the application of testing therapeutics developed for neurodegenerative and neuropsychiatric disorders with associated cognitive deficits is discussed further in Section 5.

## 3. ERP P300 Methodology

ERP P300 is a large positive waveform (normal amplitudes of up to 10–20 *μ*V) that typically occurs with a latency, or time to peak, that ranges from 250 to 500 ms poststimulus onset [3]. Recommendations for standardizing the ERP P300 methodology have been made previously [23]. Electrodes for recording ERP P300 often include three midline electrode sites—frontal, central, and parietal—with the remaining electrodes used as a reference, monitoring of the electrooculogram for eye movement and blinks, or for better definition of scalp voltage and current maps. An earlobe is often used for a reference electrode, and linked mastoids or noncephalic references can also be used effectively. ERP P300 can measure brain responses to any type of stimulus (i.e., auditory, visual, and somatosensory). P300 latency increases with the difficulty in distinguishing between stimuli. The oddball task (described further in Section 4) is the most common paradigm used for measuring the P300; however, other tasks have also been used, including the continuous performance task, the Eriksen flanker task, the Stroop task, [23], and the sustained attention to response task, or go/no-go task [26].

When comparing results of the ERP P300 across the literature, certain methodological factors should be considered, including test-retest variability. It is possible to reduce test-retest variability by increasing the signal-to-noise ratio and by recording a sufficient prestimulus baseline. To increase the signal-to-noise ratio, ERP P300 recordings are generally repeated over several (35–60) experimental trials, and an averaged representative waveform with several components is produced [27]. Recommendations provided in Duncan et al. [23] include utilizing a baseline of 100–150 ms prior to a stimulus and 800–1000 ms after a stimulus in each epoch [23]. Recording of a sufficient baseline allows distinguishing between normal fluctuations within a subject's response that can be later subtracted from the poststimulus response during analysis [27]. Using varying interstimulus intervals helps reduce stimulus predictability and some anticipatory brain potentials (e.g., contingent negative variation and lateralized readiness potential). Additionally, using the proper band-pass can filter out frequencies to reduce noise without affecting signal; however, it is recommended that filtering take place during analysis rather than during recording [23, 27].

Methods to reduce recording noise in the analysis phase include subtracting the baseline from the poststimulus signal. Another method used to reduce noise is to average the experimental trials for one subject to produce a representative waveform for that subject, excluding trials with incorrect responses. A further step often employed entails averaging the waveform for all subjects within a group to produce a grand average that can be used for comparison. Most studies use grand averaged waveforms for data presentation. However, the grand average does not capture trial-to-trial differences; similarly, the group grand average does not capture subject-to-subject variability (which can be added by shading an area of ±1 standard error, standard deviation, or other variability metric). At least one study has reported that the independent component analysis of as few as five trials can differentiate between groups of subjects [28].

## 4. ERP P300 Auditory Oddball Paradigm

The oddball paradigm is a discrimination task that requires working memory to decipher between a standard stimulus and an infrequent stimulus. This task is the most used technique to evaluate changes in cognition using the ERP P300 measurement, and it can be performed with visual or auditory stimuli. Several considerations must be taken in the design of the oddball task (i.e., stimulus relevance, probability, distractibility, and focused attention) as they can affect cognitive response. For example, an individual's arousal state can be impacted by the stimulus presented and the subsequent response [29]. The difficulty of the task can also affect the results: for example, an easier task may not discriminate between groups, while a more difficult task may be able to detect differences between the same subject groups [30].

An auditory oddball task paradigm consists of the presentation of audio tones, including frequent standard tones (e.g., 85% low-pitch [500-Hz]) and rare oddball (e.g., 15% high-pitch [2000-Hz]) tones with relatively short (e.g., 1.2 s to 1.9 s) interstimulus intervals. Over the course of the task, participants are required to count the number of oddball tones they hear; therefore, it is important to conduct prior testing to demonstrate adequate hearing capabilities for the frequencies utilized. The P300 deflection is evoked within a typical range of 250–500 ms after the presentation of the oddball tone, with a positive voltage peak occurring normally at approximately 300 ms after the end of the odd tone [3, 29]. A similar peak is not seen when the standard tone is presented as this tone is not being actively stored in working memory; in contrast, both types of tones elicit N1, P2, and N2 activity, which are associated with sensory and automatic attentional processes.

Counting of the oddball tones may be recorded mentally, or participants may be asked to respond to each target tone by pressing a button. While the use of the button-press allows for the recording of both accuracy and reaction time to correlate with P300 latency and may help maintain engagement with the task, evidence suggests that its use may introduce movement-related artifacts (and lateralized readiness potentials) and may reduce the amplitude of the P300 [31]. Mental recording of the response allows participants to focus on the accuracy of their response rather than on responding as quickly as possible. However, while reaction times have been shown to increase in patients with AD as compared to healthy age-matched controls, accuracy did not differ between the groups [32]. Additional evidence suggests that mental count may require additional working memory capacity; therefore, altering the P300 waveform [33] and the frontal P300 produced may also be an indicator of frontal-executive abilities [34].

## 5. Neuroanatomical Substrates of the P300

Human lesion studies and intracranial studies demonstrate temporoparietal, frontal, limbic, and paralimbic P300 generators [35]. Convergent neuroimaging evidence using functional magnetic resonance imaging (fMRI) indicates that the neural activation in brain regions surrounding the temporal-parietal junction and lateral prefrontal cortex acts as core generators of P300 [35, 36]. In addition, modality-specific activations in regions such as the superior temporal gyrus (auditory) and the occipital regions (visual) are also involved in a task-specific manner [35]. Neuroanatomical substrates of P300, as shown previously within, comprise prominent nodes of major functional brain networks such as the frontoparietal attentional control network, saliency network, and default mode network [37]. Disruption of these networks impairs cognitive function, including attention, working memory, and episodic memory, and has important impact on cognition in neurological and psychiatric disorders such as AD, depression, and schizophrenia [37]. Specific to age-related neurodegeneration, brain areas of convergent age- and AD-related atrophy are present in the parietal angular gyrus and dorsolateral prefrontal cortex [38], which are core neural generators of P300.

## 6. ERP in Dementia

Numerous studies have used the ERP to study cognition in various neurodegenerative, neuropsychiatric, and neurodevelopmental disorders. Several studies have detected changes in the early ERP waveform components associated with sensory processes in patients with AD. Longer latencies of several components of the ERP have been reported in patients with AD, as well as family members carrying genetic mutations related to AD [39]. Hirata et al. [40] reported a decreased global field power of the N100 in an oddball paradigm, while Tarkka et al. [41] showed decreased N100 peak amplitude and latency in patients with familial AD compared with healthy controls when performing a habituation task [40, 41]. Longer latencies of both P200 and N200 in the two-back task in patients with AD have also been reported [42]. Additional studies have evaluated late ERP waveforms, such as the LPC/P600 and N400 measurements to evaluate cognition in patients with AD. For example, studies of patients with mild cognitive impairment (MCI) that converted to AD showed reduced or absent LPC/P600 word repetition or N400 effects prior to conversion [18, 43].

### 6.1. ERP P300 in AD

**Figure 3:**
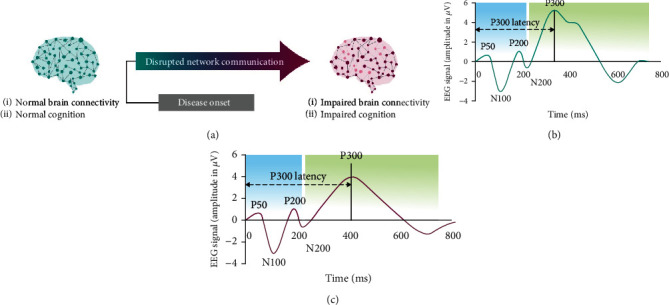
Schematic of the ERP P300 waveform in the progression of AD. Shown is a representative ERP waveform for neurotypical brain activity and normal cognition (b). A characteristic response is detected 300 ms after presentation of stimuli (P300). Early in disease onset, and usually prior to the diagnosis of AD, neural network communication is disrupted, leading to impaired brain connectivity and impaired cognition. This change in cognition can be detected as a change in the peak and/or latency (time to onset of peak) of the P300 response to stimuli (c).

While several ERP components are altered in patients with AD, the ERP P300 latency is a particularly useful tool for measuring synaptic function [6, 44–46]; the usefulness of ERP P300 in AD stems from the fact that it is only elicited when working memory is active, the simplicity of the task employed in order to evoke the response, and the size of the response in comparison to other waveforms of the ERP. Furthermore, the ERP P300 has been used for several decades to detect cognitive changes in dementia [44]. Therefore, a large body of literature is available for standardization and comparison. In diseases in which there are deficits in working memory, P300 amplitude would be expected to decrease and P300 latency would be expected to increase with disease progression (Figure 3).

ERP P300 can be used for the early assessment of cognitive decline in patients with AD. Indeed, numerous studies have shown abnormalities/differences in P300 amplitude and latency in patients with both MCI and AD [47–49]. Furthermore, P300 studies have been able to sensitively track progression of MCI and AD dementia over time [50–53]. Several studies have also shown that ERP P300 can detect differences in P300 latency in people with a family history of AD as compared to age-matched controls [39, 54–56]. Additionally, results of several meta-analyses of ERP P300 latency studies in patients with AD and MCI support the use of ERP P300 as a biological marker for prodromal AD [57–61]. While most studies have reported a decreased P300 amplitude in patients with AD [62], there have been some exceptions, in which amplitude in patients with AD was reported as being equivalent to that in healthy controls [53, 63]. In contrast, increased P300 latency has been consistently supported in the AD literature (Table 1). Although at least one study found no difference in P300 latency between patients with AD and healthy controls [64], notably, no studies have reported decreased latency in patients with AD compared with healthy controls.

ERP P300 latency is sensitive to drug effects on cognitive performance, highlighting its utility as a biomarker in early clinical trials. As early as 1987, ERP P300 was used to assess the efficacy of treatment in patients with AD; in a double-blind crossover study, an oral muscarinic agonist improved ERP P300 amplitude compared with placebo [65]. Nicergoline (an ergoline derivative) administration resulted in reduced P300 latency in patients with dementia [66, 67]. Treatment with donepezil and rivastigmine (acetylcholinesterase inhibitors) also resulted in reduced P300 latency and improved cognitive scores in patients with AD [68–72]. A recent randomized trial showed an increase in amplitude of the ERP P300 in patients with cognitive impairment after treatment with HTL0009936 (a selective muscarinic M_1_-acetylcholine receptor agonist) compared to treatment with placebo [73]. Most recently, a double-blind, placebo-controlled phase 1 clinical trial showed a reduction in P300 latency in patients with AD treated with fosgonimeton (a hepatocyte growth factor (HGF)/MET–positive modulator) compared with placebo [74]. Additionally, an ongoing randomized, double-blind, 26-week phase 2 trial (ACT-AD, NCT04491006) in patients with mild to moderate AD is using the change in P300 latency as the primary endpoint in assessing the effects of fosgonimeton. Another ongoing randomized, double-blind, placebo-controlled phase 1 trial (NCT04759365) is measuring the change in P300 latency to assess ASN51 (an oral, small molecule inhibitor of (protein) 3-O-(N-acetyl-D-glucosaminyl)-L-serine/threonine N-acetylglucosaminyl hydrolase) in healthy subjects and patients with AD.

Beyond drugs that specifically target AD, the antihypertensive valsartan improved cognitive scores and reduced the P300 latency [75]. Administration of modafinil (a wakefulness agent) in subjects with narcolepsy or idiopathic hypersomnia improved P300 latency and cognition compared with placebo [76, 77]. In addition to the evidence for restoration of P300 latency by therapeutics, the contrary has also been demonstrated with the muscarinic receptor antagonist scopolamine. Scopolamine has been used to model cognitive deficits and has been shown to increase visual P300 latency while reducing cognitive performance [78, 79].

### 6.2. ERP P300 in Other Neurological Disorders and Neuropsychiatric Disorders

Additional utility of ERP P300 has been noted in the studies of neurodegenerative disorders other than AD. For example, Parkinson's disease dementia (PDD) and dementia with Lewy bodies (DLB) are associated with increased P300 latency. In one study, patients with DLB showed more severe delayed P300 latency on an auditory oddball paradigm than did patients with AD [80]. Both auditory [81–83] and visual [84] paradigms have been used to evaluate cognition in patients with PDD and have demonstrated increased P300 latency [81–83] and reduced amplitudes [83, 84] as compared to age-matched healthy controls. Furthermore, ERP P300 may also be a useful tool in diagnosing MCI in patients with PD [85, 86]. While several studies have utilized ERP P300 to study PDD and DLB, we are unaware of any clinical trials that have used ERP P300 to detect drug-induced changes in cognition for PDD or DLB to date. An ongoing randomized, double-blind, placebo-controlled, parallel-group, 26-week phase 2 trial (SHAPE, NCT04831281) that will evaluate changes in cognition and ERP P300 latency in patients with mild to moderate PDD or DLB treated with fosgonimeton or placebo is the first controlled clinical trial that includes this outcome measure.

A wide range of studies have utilized the P300 in patients with multiple sclerosis. These include studies on cognitive impairment and fatigue [87], deep gray matter atrophy association with cognition [88], cognition during relapse [89], and the ability of ERP P300 to predict long-term disability [90]. Also, the use of ERP P300 has been suggested as a complementary tool to correlate with cognitive scores in patients with epilepsy [91]. A recent meta-analysis showed that patients with epilepsy have longer P300 latency and lower P300 amplitude than controls [92]. Meador and colleagues' study had a large influence on clinical practice when it demonstrated prolonging of P300 latency with antiepileptic drugs [93]. Indeed, the prolonged P300 latency has also been demonstrated in other indications with working memory deficits, including HD [26], transient ischemic stroke [94], intellectual disability [25], attention deficit hyperactivity disorder [95], and sleep deprivation [96, 97]. Additionally, evaluation of ERP P300 in patients with TBI demonstrated increased P300 latency and reduced amplitude compared with healthy controls [24, 98]. Furthermore, evidence from a study of military service members supported P300 latency change as a quantitative biomarker for monitoring mental health changes following trauma, including posttraumatic stress disorder, depression, and psychosocial functioning [99].

While other components of the ERP are often used to assess various aspects of neuropsychiatric disorders, reviewed by Sur and Sinha [15], the ability of the P300 component to predict drug effects on cognition is also relevant to drug development for mood disorders regularly associated with cognitive impairment, such as major depressive disorder (MDD) and schizophrenia. Prolonged P300 latency and cognitive deficits have been demonstrated in patients with MDD [100], and P300 latency is directly proportional to MDD severity [101, 102]; these findings support the use of P300 latency as a prognostic indicator for and potential measure of therapeutic response to antidepressant treatment. A recent study of the N-menthyl-D-aspartate (NMDA) receptor antagonist ketamine used healthy subjects and ERP P300 to evaluate its potential utility for schizophrenia research [103]. A systematic review recently reported that the increased latency and reduced amplitude of the P300 are common findings in the early stages of schizophrenia, which lends support for the concept that synaptic dysfunction precedes the onset of severe symptoms, akin to the neuropathology of neurodegenerative diseases [104].

## 7. Conclusion

ERP P300 assessments can directly measure large neuronal network functioning in distinct settings (e.g., in response to auditory stimuli) and can answer a spectrum of important questions in early procognitive drug development. ERP P300 assessment offers several advantages over the current practice of measuring certain brain protein concentrations as a potentially predictive biomarker for cognitive outcomes [11]. The ability to extrapolate results of ERP P300 assessment early on in drug development lends support for rational target dose range decisions and increases confidence in staging larger and longer controlled clinical trials. The utility of ERP P300 latency in studying the progression of cognitive decline in patients with AD has been consistently supported in the literature. Additionally, ERP P300 latency has potential uses in many other neurological and psychiatric disorders. Importantly, P300 latency is an ideal measure that is sensitive to change in cognitive processing occurring in both, disease progression in AD, and in response to drug treatments over short durations. Overall, the evidence reviewed here supports the use of ERP P300 latency as an objective and noninvasive surrogate biomarker for predicting the therapeutic potential of drugs in clinical development for neurocognitive disorders.

## Figures and Tables

**Table 1:** ERP P300 studies in patients with AD.

Study	Population (*n*)	P300 latency	P300 amplitude
Ally et al., [54]	HC (80) AD (80)	AD > HC	HC > AD
Bennys et al., [47]	HC (10) MCI (20) AD (30)	AD > MCI > HC	HC > MCI = AD
Caravaglios et al., [63]	HC (16) AD (21)	AD > HC	HC = AD
Cecchi et al., [105]	HC (101) Mild AD (103)	AD > HC	HC > AD
Cintra et al., [106]	HC (14) MCI (34) AD (17)	(ApoE4+) MCI + AD > HC	HC = AD
Frodl et al., [48]	HC (26) MCI (26) AD (30)	AD > MCI > HC	HC > MCI = AD
Fruehwirt et al., [107]	Mild AD (31) Severe AD (32)	Severe > mild	NA
Golob and Starr, [32]	HC (12) AD (10)	AD > HC	HC > AD
Hirata et al., [40]	HC (12) AD (26)	AD > HC	HC > AD
Jervis et al., [28, 108–110]	HC (9) AD (9)	AD > HC	HC = AD
Juckel et al., [111]	HC (18) AD (18)	AD > HC	HC > AD
Kraiuhin et al., [112]	HC (100) AD (25)	AD > HC (but not statistically significant)	NA
Lai et al., [53]	HC (16) AD (16)	AD > HC	HC = AD
Lee et al., [64]	HC (31) AD (31)	AD = HC	HC > AD
Marsh et al., [113]	HC (17) AD (18)	AD > HC	NA
O'Mahony et al., [114]	HC (20) AD (18)	AD > HC	NA
Papadaniil et al., [115]	HC (21) MCI (21) AD (21)	AD > MCI > HC	HC > MCI > AD (but not statistically significant)
Papaliagkas et al., [116]	HC (30) MCI (49) AD (5)	AD, MCI > HC	HC = MCI, AD
Papaliagkas, et al., [117]	MCI (15) AD (5)	AD > MCI	MCI > AD
Pedroso et al., [118]	HC (30) Mild AD (24)	AD > HC	NA
Pedroso et al., [119]	HC (37) AD (48)	AD > HC	HC > AD
Pokryszko-Dragan et al., [120]	HC (13) AD (13)	AD > HC	HC > AD
Polich et al., [121]	HC (16) AD (16)	AD > HC	HC > AD
Polich and Corey-Bloom, [30]	HC (NA) AD (NA)	AD > HC	HC > AD
Tachibana et al., [122]	HC (15) AD (15)	AD > HC	NA
Yamaguchi et al., [123]	HC (16) AD (16)	AD > HC	HC > AD

Abbreviations: AD: Alzheimer's disease; ApoE4: apolipoprotein E4; HC: healthy control; MCI: mild cognitive impairment; and NA: not available.

## Abbreviations

AD: Alzheimer's disease
ApoE4: Apolipoprotein E4
CNS: Central nervous system
DLB: Dementia with Lewy bodies
EEG: Electroencephalography
EPs: Evoked potentials
ERPs: Event-related potentials
fMRI: Functional magnetic resonance imaging
HD: Huntington's disease
HGF: Hepatocyte growth factor
MCI: Mild cognitive impairment
MDD: Major depressive disorder
NMDA: N-methyl-D-aspartate
PD: Parkinson's disease
PDD: Parkinson's disease dementia
qEEG: Quantitative electroencephalography
REM: Rapid eye movement
TBI: Traumatic brain injury.

## References

[1] World Health Organization (2021). World Health Organization-dementia. *Home/Newsroom/Fact sheets/Detail/Dementia*.

[2] de Aquino C. H. (2021). Methodological issues in randomized clinical trials for prodromal Alzheimer's and Parkinson's disease. *Frontiers in Neurology*.

[3] Horvath A., Szucs A., Csukly G., Sakovics A., Stefanics G., Kamondi A. (2018). EEG and ERP biomarkers of Alzheimer’s disease a critical review. *Frontiers in Bioscience*.

[4] Jack C. R., Knopman D. S., Jagust W. J. (2010). Hypothetical model of dynamic biomarkers of the Alzheimer's pathological cascade. *Lancet Neurology*.

[5] Katsuno M., Sahashi K., Iguchi Y., Hashizume A. (2018). Preclinical progression of neurodegenerative diseases. *Nagoya Journal of Medical Science*.

[6] Raskin J., Cummings J., Hardy J., Schuh K., Dean R. A. (2015). Neurobiology of Alzheimer's disease: integrated molecular, physiological, anatomical, biomarker, and cognitive dimensions. *Current Alzheimer Research*.

[7] Cummings J. (2018). Lessons learned from Alzheimer disease: clinical trials with negative outcomes. *Clinical and Translational Science*.

[8] Yaari R., Hake A. (2015). Alzheimer’s disease clinical trials: past failures and future opportunities. *Clinical Investigation*.

[9] Mehta D., Jackson R., Paul G., Shi J., Sabbagh M. (2017). Why do trials for Alzheimer's disease drugs keep failing? A discontinued drug perspective for 2010-2015. *Expert Opinion on Investigational Drugs*.

[10] Mohs R. C., Greig N. H. (2017). Drug discovery and development: role of basic biological research. *Alzheimer's & Dementia: Translational Research & Clinical Interventions*.

[11] Cedazo-Minguez A., Winblad B. (2010). Biomarkers for Alzheimer's disease and other forms of dementia: clinical needs, limitations and future aspects. *Experimental Gerontology*.

[12] Babiloni C., Blinowska K., Bonanni L. (2020). What electrophysiology tells us about Alzheimer's disease: a window into the synchronization and connectivity of brain neurons. *Neurobiology of Aging*.

[13] Sokhadze E. M., Casanova M. F., Casanova E. L., Lamina E., Kelly D. P., Khachidze I. (2017). Event-related potentials (ERP) in cognitive neuroscience research and applications. *NeuroRegulation*.

[14] Leiser S. C., Dunlop J., Bowlby M. R., Devilbiss D. M. (2011). Aligning strategies for using EEG as a surrogate biomarker: a review of preclinical and clinical research. *Biochemical Pharmacology*.

[15] Sur S., Sinha V. K. (2009). Event-related potential: an overview. *Industrial Psychiatry Journal*.

[16] Olincy A., Martin L. (2005). Diminished suppression of the P50 auditory evoked potential in bipolar disorder subjects with a history of psychosis. *The American Journal of Psychiatry*.

[17] Schulze K. K., Hall M. H., McDonald C. (2007). P50 auditory evoked potential suppression in bipolar disorder patients with psychotic features and their unaffected relatives. *Biological Psychiatry*.

[18] Olichney J. M., Taylor J. R., Gatherwright J. (2008). Patients with MCI and N400 or P600 abnormalities are at very high risk for conversion to dementia. *Neurology*.

[19] Olichney J. M., Van Petten C., Paller K. A., Salmon D. P., Iragui V. J., Kutas M. (2000). Word repetition in amnesia. Electrophysiological measures of impaired and spared memory. *Brain*.

[20] Xia J., Mazaheri A., Segaert K. (2020). Event-related potential and EEG oscillatory predictors of verbal memory in mild cognitive impairment. *Brain Communications*.

[21] Polich J., Criado J. R. (2006). Neuropsychology and neuropharmacology of P3a and P3b. *International Journal of Psychophysiology*.

[22] Friedman D., Luck S. J., Kappenman E. S. (2012). The components of aging. *The Oxford Handbook of Event-Related Potential Components*.

[23] Duncan C. C., Barry R. J., Connolly J. F. (2009). Event-related potentials in clinical research: guidelines for eliciting, recording, and quantifying mismatch negativity, P300, and N400. *Clinical Neurophysiology*.

[24] Nandrajog P., Idris Z., Azlen W. N., Liyana A., Abdullah J. M. (2017). The use of event-related potential (P300) and neuropsychological testing to evaluate cognitive impairment in mild traumatic brain injury patients. *Asian Journal of Neurosurgery*.

[25] Vaney N., Khaliq F., Anjana Y. (2015). Event-related potentials study in children with borderline intellectual functioning. *Indian Journal of Psychological Medicine*.

[26] Hart E. P., Dumas E. M., van Zwet E. W. (2015). Longitudinal pilot-study of sustained attention to response task and P300 in manifest and pre-manifest Huntington's disease. *Journal of Neuropsychology*.

[27] Woodman G. F. (2010). A brief introduction to the use of event-related potentials in studies of perception and attention. *Attention, Perception, & Psychophysics*.

[28] Jervis B. W., Bigan C., Jervis M. W., Besleaga M. (2019). New-onset Alzheimer's disease and normal subjects 100% differentiated by P300. *American Journal of Alzheimer's Disease and Other Dementias*.

[29] Polich J., Kok A. (1995). Cognitive and biological determinants of P300: an integrative review. *Biological Psychology*.

[30] Polich J., Corey-Bloom J. (2005). Alzheimer's disease and P300: review and evaluation of task and modality. *Current Alzheimer Research*.

[31] Salisbury D. F., Rutherford B., Shenton M. E., McCarley R. W. (2001). Button-pressing affects P300 amplitude and scalp topography. *Clinical Neurophysiology*.

[32] Golob E. J., Starr A. (2000). Effects of stimulus sequence on event-related potentials and reaction time during target detection in Alzheimer's disease. *Clinical Neurophysiology*.

[33] Kayser J., Tenke C. E., Gil R., Bruder G. E. (2010). ERP generator patterns in schizophrenia during tonal and phonetic oddball tasks: effects of response hand and silent count. *Clinical EEG and Neuroscience*.

[34] Yang J. C., Chan S. H., Khan S. (2013). Neural substrates of executive dysfunction in fragile X-associated tremor/ataxia syndrome (FXTAS): a brain potential study. *Cerebral Cortex*.

[35] Soltani M., Knight R. T. (2000). Neural origins of the P300. *Critical Reviews in Neurobiology*.

[36] Polich J., Luck S. J., Kappenman E. S. (2012). Neuropsychology of P300. *The Oxford Handbook of Event-Related Potential Components*.

[37] Menon V. (2011). Large-scale brain networks and psychopathology: a unifying triple network model. *Trends in Cognitive Sciences*.

[38] Bakkour A., Morris J. C., Wolk D. A., Dickerson B. C. (2013). The effects of aging and Alzheimer's disease on cerebral cortical anatomy: specificity and differential relationships with cognition. *NeuroImage*.

[39] Golob E. J., Ringman J. M., Irimajiri R. (2009). Cortical event-related potentials in preclinical familial Alzheimer disease. *Neurology*.

[40] Hirata K., Hozumi A., Tanaka H. (2000). Abnormal information processing in dementia of Alzheimer type. A study using the event-related potential's field. *European Archives of Psychiatry and Clinical Neuroscience*.

[41] Tarkka I. M., Lehtovirta M., Soininen H., Pääkkönen A., Karhu J., Partanen J. (2002). Auditory adaptation is differentially impaired in familial and sporadic Alzheimer's disease. *Biomedicine & Pharmacotherapy*.

[42] Missonnier P., Deiber M. P., Gold G. (2007). Working memory load-related electroencephalographic parameters can differentiate progressive from stable mild cognitive impairment. *Neuroscience*.

[43] Olichney J. M., Iragui V. J., Salmon D. P., Riggins B. R., Morris S. K., Kutas M. (2006). Absent event-related potential (ERP) word repetition effects in mild Alzheimer's disease. *Clinical Neurophysiology*.

[44] Goodin D. S., Squires K. C., Starr A. (1978). Long latency event-related components of the auditory evoked potential in dementia. *Brain*.

[45] Jackson J., Jambrina E., Li J. (2019). Targeting the synapse in Alzheimer's disease. *Frontiers in Neuroscience*.

[46] Selkoe D. J. (2002). Alzheimer's disease is a synaptic failure. *Science*.

[47] Bennys K., Portet F., Touchon J., Rondouin G. (2007). Diagnostic value of event-related evoked potentials N200 and P300 subcomponents in early diagnosis of Alzheimer's disease and mild cognitive impairment. *Journal of Clinical Neurophysiology*.

[48] Frodl T., Meisenzahl E. M., Muller D. (2002). P300 subcomponents and clinical symptoms in schizophrenia. *International Journal of Psychophysiology*.

[49] Morrison C., Rabipour S., Knoefel F., Sheppard C., Taler V. (2018). Auditory event-related potentials in mild cognitive impairment and Alzheimer's disease. *Current Alzheimer Research*.

[50] Ball S. S., Marsh J. T., Schubarth G., Brown W. S., Strandburg R. (1989). Longitudinal P300 latency changes in Alzheimer's disease. *Journal of Gerontology*.

[51] Bennys K., Rondouin G., Benattar E., Gabelle A., Touchon J. (2011). Can event-related potential predict the progression of mild cognitive impairment?. *Journal of Clinical Neurophysiology*.

[52] Golob E. J., Irimajiri R., Starr A. (2007). Auditory cortical activity in amnestic mild cognitive impairment: relationship to subtype and conversion to dementia. *Brain*.

[53] Lai C. L., Lin R. T., Liou L. M., Liu C. K. (2010). The role of event-related potentials in cognitive decline in Alzheimer's disease. *Clinical Neurophysiology*.

[54] Ally B. A., Jones G. E., Cole J. A., Budson A. E. (2006). The P300 component in patients with Alzheimer's disease and their biological children. *Biological Psychology*.

[55] Boutros N., Torello M. W., Burns E. M., Wu S. S., Nasrallah H. A. (1995). Evoked potentials in subjects at risk for Alzheimer's disease. *Psychiatry Research*.

[56] Green J., Levey A. I. (1999). Event-related potential changes in groups at increased risk for Alzheimer disease. *Archives of Neurology*.

[57] Howe A. S., Bani-Fatemi A., De Luca V. (2014). The clinical utility of the auditory P300 latency subcomponent event-related potential in preclinical diagnosis of patients with mild cognitive impairment and Alzheimer's disease. *Brain and Cognition*.

[58] Jiang S., Qu C., Wang F. (2015). Using event-related potential P300 as an electrophysiological marker for differential diagnosis and to predict the progression of mild cognitive impairment: a meta-analysis. *Neurological Sciences*.

[59] Paitel E. R., Samii M. R., Nielson K. A. (2021). A systematic review of cognitive event-related potentials in mild cognitive impairment and Alzheimer's disease. *Behavioural Brain Research*.

[60] Pedroso R. V., Fraga F. J., Corazza D. I. (2012). P300 latency and amplitude in Alzheimer's disease: a systematic review. *Brazilian Journal of Otorhinolaryngology*.

[61] Tarawneh H. Y., Mulders W. H., Sohrabi H. R., Martins R. N., Jayakody D. M. (2021). Investigating auditory electrophysiological measures of participants with mild cognitive impairment and Alzheimer's disease: a systematic review and meta-analysis of event-related potential studies. *Journal of Alzheimer's Disease*.

[62] Hedges D., Janis R., Mickelson S., Keith C., Bennett D., Brown B. L. (2016). P300 amplitude in Alzheimer's disease. *Clinical EEG and Neuroscience*.

[63] Caravaglios G., Costanzo E., Palermo F., Muscoso E. G. (2008). Decreased amplitude of auditory event-related delta responses in Alzheimer's disease. *International Journal of Psychophysiology*.

[64] Lee M. S., Lee S. H., Moon E. O. (2013). Neuropsychological correlates of the P300 in patients with Alzheimer's disease. *Progress in Neuro-Psychopharmacology & Biological Psychiatry*.

[65] Hollander E., Davidson M., Mohs R. C. (1987). RS 86 in the treatment of Alzheimer's disease: cognitive and biological effects. *Biological Psychiatry*.

[66] Iwanami A., Fujishima T., Iritani S., Makino Y., Suga I., Ikeda K. (1993). Effects of nicergoline on the P300 component of event-related potentials in demented patients. *Neurology, Psychiatry and Brain Research*.

[67] Saletu B., Paulus E., Linzmayer L. (1995). Nicergoline in senile dementia of Alzheimer type and multi-infarct dementia: a double-blind, placebo-controlled, clinical and EEG/ERP mapping study. *Psychopharmacology*.

[68] Chang Y. S., Chen H. L., Hsu C. Y., Tang S. H., Liu C. K. (2014). Parallel improvement of cognitive functions and P300 latency following donepezil treatment in patients with Alzheimer's disease: a case-control study. *Journal of Clinical Neurophysiology*.

[69] Katada E., Sato K., Sawaki A., Dohi Y., Ueda R., Ojika K. (2003). Long-term effects of donepezil on P300 auditory event-related potentials in patients with Alzheimer's disease. *Journal of Geriatric Psychiatry and Neurology*.

[70] Paci C., Gobbato R., Carboni T., Sanguigni S., Santone A., Curatola L. (2006). P300 auditory event-related potentials and neuropsychological study during donepezil treatment in vascular dementia. *Neurological Sciences*.

[71] Thomas A., Iacono D., Bonanni L., D'Andreamatteo G., Onofrj M. (2001). Donepezil, rivastigmine, and vitamin E in Alzheimer disease: a combined P300 event-related potentials/neuropsychologic evaluation over 6 months. *Clinical Neuropharmacology*.

[72] Werber A. E., Klein C., Rabey J. M. (2001). Evaluation of cholinergic treatment in demented patients by P300 evoked related potentials. *Neurologia i Neurochirurgia Polska*.

[73] Bakker C., van der Aart J., Labots G. (2021). Safety and pharmacokinetics of HTL0018318, a novel M1 receptor agonist, given in combination with donepezil at steady state: a randomized trial in healthy elderly subjects. *Drugs in R&D*.

[74] Hua X., Church K., Walker W. (2022). Safety, tolerability, pharmacokinetics, and pharmacodynamics of the positive modulator of HGF/MET, fosgonimeton, in healthy volunteers and subjects with Alzheimer's disease: randomized, placebo-controlled, double-blind, Phase I Clinical Trial. *Journal of Alzheimer's Disease*.

[75] Katada E., Uematsu N., Takuma Y., Matsukawa N. (2014). Comparison of effects of valsartan and amlodipine on cognitive functions and auditory p300 event-related potentials in elderly hypertensive patients. *Clinical Neuropharmacology*.

[76] Saletu M., Anderer P., Saletu-Zyhlarz G. M., Mandl M., Saletu B., Zeitlhofer J. (2009). Modafinil improves information processing speed and increases energetic resources for orientation of attention in narcoleptics: double-blind, placebo- controlled ERP studies with low-resolution brain electromagnetic tomography (LORETA). *Sleep Medicine*.

[77] Yaman M., Karakaya F., Aydin T., Mayda H., Güzel H. İ., Kayaalp D. (2015). Evaluation of the effect of Modafinil on cognitive functions in patients with idiopathic hypersomnia with P300. *Medical Science Monitor*.

[78] Meador K. J., Loring D. W., Adams R. J., Patel B. R., Davis H. C., Hammond E. J. (1987). Central cholinergic systems and the P3 evoked potential. *The International Journal of Neuroscience*.

[79] Potter D. D., Pickles C. D., Roberts R. C., Rugg M. D. (2000). Scopolamine impairs memory performance and reduces frontal but not parietal visual P3 amplitude. *Biological Psychology*.

[80] Bonanni L., Franciotti R., Onofrj V. (2010). Revisiting P300 cognitive studies for dementia diagnosis: Early dementia with Lewy bodies (DLB) and Alzheimer disease (AD). *Neurophysiologie Clinique*.

[81] Folmer R. L., Vachhani J. J., Riggins A. (2021). Electrophysiological evidence of auditory and cognitive processing deficits in Parkinson disease. *BioMed Research International*.

[82] Romagnolo A., Zibetti M., Lenzi M. (2021). Low frequency subthalamic stimulation and event-related potentials in Parkinson disease. *Parkinsonism & Related Disorders*.

[83] Tokic K., Titlic M., Beganovic-Petrovic A., Suljic E., Romac R., Silic S. (2016). P300 wave changes in patients with Parkinson's disease. *Medical Archives*.

[84] Ozmus G., Yerlikaya D., Gokceoglu A. (2017). Demonstration of early cognitive impairment in Parkinson's disease with visual P300 responses. *Noro Psikiyatri Arsivi*.

[85] Hünerli D., Emek-Savaş D. D., Çavuşoğlu B., Çolakoğlu B. D., Ada E., Yener G. G. (2019). Mild cognitive impairment in Parkinson's disease is associated with decreased P300 amplitude and reduced putamen volume. *Clinical Neurophysiology*.

[86] Yilmaz F. T., Özkaynak S. S., Barçin E. (2017). Contribution of auditory P300 test to the diagnosis of mild cognitive impairment in Parkinson's disease. *Neurological Sciences*.

[87] Paolicelli D., Manni A., Iaffaldano A. (2021). Magnetoencephalography and high-density electroencephalography study of acoustic event related potentials in early stage of multiple sclerosis: a pilot study on cognitive impairment and fatigue. *Brain Sciences*.

[88] Lorefice L., Fenu G., Mammoliti R. (2021). Event-related potentials and deep grey matter atrophy in multiple sclerosis: exploring the possible associations with cognition. *Multiple Sclerosis and Related Disorders*.

[89] Gedizlioglu M., Koskderelioglu A., Vural M., Tiftikcioglu I. B. (2021). Cognition in acute relapses: a psychometric evaluation and its correlation with event-related potential, P300 in multiple sclerosis. *Applied Neuropsychology: Adult*.

[90] Szilasiová J., Rosenberger J., Mikula P., Vitková M., Fedičová M., Gdovinová Z. (2020). Cognitive event-related potentials–the P300 wave is a prognostic factor of long-term disability progression in patients with multiple sclerosis. *Journal of Clinical Neurophysiology*.

[91] Mukheem Mudabbir M. A., Mundlamuri R. C., Aravind K. R. (2021). EEG-based P300 in mesial temporal lobe epilepsy and its correlation with cognitive functions: a case-control study. *Epilepsy & Behavior*.

[92] Zhong R., Li M., Chen Q., Li J., Li G., Lin W. (2019). The P300 event-related potential component and cognitive impairment in epilepsy: a systematic review and meta-analysis. *Frontiers in Neurology*.

[93] Meador K. J., Loring D. W., Huh K., Gallagher B. B., King D. W. (1990). Comparative cognitive effects of anticonvulsants. *Neurology*.

[94] Zhang Y., Xu H., Zhao Y., Zhang L., Zhang Y. (2021). Application of the P300 potential in cognitive impairment assessments after transient ischemic attack or minor stroke. *Neurological Research*.

[95] Kim J. S., Lee Y. J., Shim S. H. (2021). What event-related potential tells us about brain function: child-adolescent psychiatric perspectives. *Soa Chongsonyon Chongsin Uihak*.

[96] Choudhary A. K., Kishanrao S. S., Dadarao Dhanvijay A. K., Alam T. (2016). Sleep restriction may lead to disruption in physiological attention and reaction time. *Sleep Science*.

[97] Lee H. J., Kim L., Suh K. Y. (2003). Cognitive deterioration and changes of P300 during total sleep deprivation. *Psychiatry and Clinical Neurosciences*.

[98] Li H., Li N., Xing Y. (2021). P300 as a potential indicator in the evaluation of neurocognitive disorders after traumatic brain injury. *Frontiers in Neurology*.

[99] Wang C., Rapp P., Darmon D. (2018). Utility of P300 ERP in monitoring post-trauma mental health: a longitudinal study in military personnel returning from combat deployment. *Journal of Psychiatric Research*.

[100] Himani A., Tandon O. P., Bhatia M. S. (1999). A study of P300-event related evoked potential in the patients of major depression. *Indian Journal of Physiology and Pharmacology*.

[101] Hetzel G., Moeller O., Evers S. (2005). The astroglial protein S100B and visually evoked event-related potentials before and after antidepressant treatment. *Psychopharmacology*.

[102] Tripathi S. M., Mishra N., Tripathi R. K., Gurnani K. C. (2015). P300 latency as an indicator of severity in major depressive disorder. *Industrial Psychiatry Journal*.

[103] de la Salle S., Shah D., Choueiry J. (2021). *N*-methyl-*d*-aspartate receptor antagonism modulates P300 event-related potentials and associated activity in salience and central executive networks. *Pharmacology, Biochemistry, and Behavior*.

[104] Perrottelli A., Giordano G. M., Brando F., Giuliani L., Mucci A. (2021). EEG-based measures in at-risk mental state and early stages of schizophrenia: a systematic review. *Frontiers in Psychiatry*.

[105] Cecchi M., Moore D. K., Sadowsky C. H. (2015). A clinical trial to validate event-related potential markers of Alzheimer's disease in outpatient settings. *Alzheimer's & Dementia: Diagnosis, Assessment & Disease Monitoring*.

[106] Cintra M. T. G., Ávila R. T., Soares T. O. (2018). Increased N200 and P300 latencies in cognitively impaired elderly carrying *ApoE ε*-4 allele. *International Journal of Geriatric Psychiatry*.

[107] Fruehwirt W., Dorffner G., Roberts S. (2019). Associations of event-related brain potentials and Alzheimer's disease severity: a longitudinal study. *Progress in Neuro-Psychopharmacology & Biological Psychiatry*.

[108] Jervis B. W., Bigan C., Besleaga M. (2020). New-onset Alzheimer's disease and normal subjects 100% separated statistically by P300 and ICA. *American Journal of Alzheimer's Disease and Other Dementias*.

[109] Jervis B., Belal S., Camilleri K. (2007). The independent components of auditory P300 and CNV evoked potentials derived from single-trial recordings. *Physiological Measurement*.

[110] Jervis B. W., Belal S., Cassar T. (2010). Waveform analysis of non-oscillatory independent components in single-trial auditory event-related activity in healthy subjects and Alzheimer's disease patients. *Current Alzheimer Research*.

[111] Juckel G., Clotz F., Frodl T. (2008). Diagnostic usefulness of cognitive auditory event-related p300 subcomponents in patients with Alzheimers disease?. *Journal of Clinical Neurophysiology*.

[112] Kraiuhin C., Gordon E., Coyle S. (1990). Normal latency of the P300 event-related potential in mild-to-moderate Alzheimer's disease and depression. *Biological Psychiatry*.

[113] Marsh J. T., Schubarth G., Brown W. S. (1990). PET and P300 relationships in early Alzheimer's disease. *Neurobiology of Aging*.

[114] O'Mahony D., Coffey J., Murphy J. (1996). Event-related potential prolongation in Alzheimer's disease signifies frontal lobe impairment: evidence from SPECT imaging. *The Journals of Gerontology Series A: Biological Sciences and Medical Sciences*.

[115] Papadaniil C. D., Kosmidou V. E., Tsolaki A., Tsolaki M., Kompatsiaris I. Y., Hadjileontiadis L. J. (2016). Cognitive MMN and P300 in mild cognitive impairment and Alzheimer's disease: a high density EEG-3D vector field tomography approach. *Brain Research*.

[116] Papaliagkas V., Kimiskidis V., Tsolaki M., Anogianakis G. (2008). Usefulness of event-related potentials in the assessment of mild cognitive impairment. *BMC Neuroscience*.

[117] Papaliagkas V. T., Anogianakis G., Tsolaki M. N., Koliakos G., Kimiskidis V. K. (2010). Combination of P300 and CSF *β*-amyloid(1-42) assays may provide a potential tool in the early diagnosis of Alzheimers disease. *Current Alzheimer Research*.

[118] Pedroso R. V., Corazza D. I., Andreatto C. A. D. A., Silva T. M. V. D., Costa J. L. R., Santos-Galduróz R. F. (2018). Cognitive, functional and physical activity impairment in elderly with Alzheimer's disease. *Dementia & Neuropsychologia*.

[119] Pedroso R. V., Fraga F. J., Nascimento C. M. C., Pott-Junior H., Cominetti M. R. (2022). *Apolipoprotein E ε4* allele impairs cortical activity in healthy aging and Alzheimer's disease. *Behavioural Brain Research*.

[120] Pokryszko-Dragan A., Słotwiński K., Podemski R. (2003). Modality-specific changes in P300 parameters in patients with dementia of the Alzheimer type. *Medical Science Monitor*.

[121] Polich J., Ladish C., Bloom F. E. (1990). P300 assessment of early Alzheimer's disease. *Electroencephalography and Clinical Neurophysiology*.

[122] Tachibana H., Takeda M., Okuda B. (1996). Multimodal evoked potentials in Alzheimer's disease and Binswanger's disease. *Journal of Geriatric Psychiatry and Neurology*.

[123] Yamaguchi S., Tsuchiya H., Yamagata S., Toyoda G., Kobayashi S. (2000). Event-related brain potentials in response to novel sounds in dementia. *Clinical Neurophysiology*.

[124] Seeley W. W., Menon V., Schatzberg A. F. (2007). Dissociable intrinsic connectivity networks for salience processing and executive control. *The Journal of Neuroscience*.

